# Foliar disease resistance phenomics of fungal pathogens: image-based approaches for mapping quantitative resistance in cereal germplasm

**DOI:** 10.1007/s00122-025-05017-4

**Published:** 2025-08-28

**Authors:** Matthew Ulrich, Linda Brain, Jianqiao Zhang, Anthony R. Gendall, Stefanie Lück, Dimitar Douchkov, Eden Tongson, Peter M. Dracatos

**Affiliations:** 1 La Trobe Institute of Sustainable Agriculture & Food (LISAF), Department of Ecological, Plant and Animal Sciences, School of Agriculture, Biomedicine and Environment, La Trobe University, Bundoora, VIC 3086 Australia; 2 Leibniz Institute of Plant Genetics and Crop Plant Research (IPK), 06466 Seeland OT Gatersleben, Germany

## Abstract

Host plant resistance is the most effective and environmentally sustainable means of reducing yield losses caused by fungal foliar pathogens of cereal species. Cereal genebank collections hold diverse pools of potentially underutilized disease resistance alleles, and cereal genomic resources are well advanced due to large-scale sequencing and genotyping efforts. Genome-Wide Association Studies (GWAS) have emerged as the predominant association genetics technique to initially discover novel disease resistance loci or alleles in these diverse collections. Traditional disease resistance phenotyping methods are reliant on visual estimation of disease symptom severity and have successfully supported genetic mapping studies either via GWAS or QTL mapping in biparental populations facilitating both marker development and gene cloning efforts. Due to foliar pathogens having a high capacity to evolve, there is a need to pyramid disease resistance genes with diverse mechanisms for durable control. Resistance expressed as a quantitative trait, known as quantitative resistance (QR), is hypothesized to be more durable, unlike major *R*-gene resistance that is race-specific and can be vulnerable to breaking down without gene stewardship. However, assessing QR visually is challenging, particularly when complicated by complex genotype × environment (*G* × *E*) effects in the field. High-throughput image-based phenotyping provides accurate and unbiased data that can support foliar disease resistance screening efforts of genebank collections using GWAS. In this review, we discuss image-based disease phenotyping based on macroscopic (visible symptoms) and microscopic features during the host–pathogen interaction. Quantitative image analysis approaches using conventional and artificial intelligence (AI) algorithms are also discussed.

## Introduction

Whole-grain cereals are grown worldwide for their nourishing carbohydrates and essential nutrients (proteins, vitamins, minerals, and phytochemicals) that help prevent diseases such as heart attacks and cancer (Aune et al. 2016). As staple foods in the human diet, these grains are of immense societal economic importance in many countries which drives continuous efforts to enhance crop productivity and resilience in the face of agricultural challenges.

Global genebanks maintain extensive collections of diverse wild and domesticated germplasm with associated passport data (accession name, genus, country of origin, date, basic description, more recently genotypic data, etc.). These plant genetic resources (PGRs) are the raw materials used for crop improvement, and PGRs are prioritized for breeding based on large-scale phenotyping and genotyping. The cereal crops wheat, rice, and barley represent the bulk of these collections with 850,000, 770,000 and 460,000 accessions stored across global genebanks, respectively (FAO 2010; Ramirez-Villegas et al. 2022). Genebanks capture 76% and 71% of the total genetic variation in barley and wheat landraces, respectively, and these PGRs are hypothesized to harbor underutilized resistance genes and alleles (Ramirez-Villegas et al. 2022). Geopolitical constraints have largely influenced the movement of germplasm, mirroring trends in human migration, and this has bottlenecked disease resistance allelic diversity, among other important agronomic traits (Arifuzzaman et al. 2023).

Fungal foliar pathogens are a constant threat to the secure production of cereal grains, and the prevalence of these pathogens is forecasted to increase in predicted climate scenarios (Garrett et al. 2011; Velásquez et al. 2018; Raza et al. 2019). Utilization of host plant resistance is the most effective, environmentally sustainable and economical method for controlling cereal foliar diseases (Niks et al. 2015; Scortichini 2022; Panthee et al. 2024). The emergence of fungicide-resistant foliar pathogens is a growing concern that underscores the need to find new sources of durable host plant resistance (Yin et al. 2023). Resistant cultivars can also reduce or eliminate reliance on costly fungicides, an attractive prospect for health conscious consumers and for growers maximizing profitability on farm.

The development of efficient strategies to mine vast collections of PGRs for underutilized resistance donors is critical to overcome current domestication bottlenecks that constrain allelic diversity and limit progress toward disease resistance breeding and food security. Genome-Wide Association Studies (GWAS) are the predominant approach to statistically model the relationship between genotype data and phenotype data using bioinformatics based on linkage disequilibrium (LD) at the population level, where larger and more diverse populations result in faster LD decay and increased power for detecting significant associations (Hill and Robertson 1968; Altshuler 2005; Desaint et al. 2023). GWAS can be used to map quantitative trait loci (QTLs) or even causal variants associated with phenotypic variation of agronomic traits in diversity panels with population structures beyond experimental crosses (Marsh et al. 2021; Desaint et al. 2023). GWAS requires 2 main inputs: (i) genotype data with sufficient marker density obtained via a SNP array or targeted genotyping-by-sequencing (GBS) approach and (ii) precise quantitative phenotypic data from a large population to enhance the detection accuracy and frequency of significant marker–trait association (MTAs) (Desaint et al. 2023; Clauw et al. 2024). SNP arrays are a popular choice among breeders and researchers, with breeders desiring low-cost/low-density SNP arrays, while researchers require high-density arrays to increase the precision of GWAS signals to narrow down the distance between the MTA and the underlying gene for sequence isolation and subsequent functional characterization (Keeble-Gagnère et al. 2021). One limitation of GWAS is that rare variants are excluded from the analysis due to minor allele frequency (MAF) quality control parameters, leaving potentially rare genes or alleles excluded from the analysis (Jayakodi et al. 2024). In this case, a biparental mapping approach is required to map the rare causal variant in a resistant by susceptible cross; however, suppressed or low rates of recombination can be problematic (Stein and Muehlbauer 2018). GWAS also requires moderate programming knowledge that can restrict the user-friendliness of this technique; however, tools have been developed to simplify data importation, subsequent visualization of results (Lück et al. 2024).

While large-scale cereal genotyping efforts address the genomic requirement for GWAS, current disease resistance phenotyping methods are reliant on visual assessment by human operators. Phenotypic accuracy using either individual or teams of raters is subject to known pitfalls such as rater fatigue, habituation to repetitive measurements, and innate differences between raters, which can affect the accuracy and precision of the measurement (Nutter Jr et al. 1993; Pethybridge and Nelson 2015). Despite these limitations, the human eye continues to play a central role in providing phenotypic information for successful gene cloning efforts, with selection bias toward qualitative major *R*-genes due to their spectacular phenotypes as Resistant (*R*) contributing large proportions of phenotypic variance in genetic mapping/QTL mapping studies (McDonald and Linde 2002; Poland et al. 2009; Kou and Wang 2010; St. Clair 2010; Mundt 2014, 2018; Karisto et al. 2018; Dracatos et al. 2023a). For example, the cereal rusts and mildew pathosystems are genetically well characterized due to detailed historical pathogenicity surveys that have relied on the keen expert eye being able to classify different resistance infection types manifesting as reduced symptom severity, hypersensitive cell death responses (HR), or complete immunity (McIntosh 1992; Niks et al. 2015; Schulthess et al. 2022b; Bapela et al. 2023).

However, not all resistance genes are equal—qualitative resistance is often pathogen race-specific, and several reports have observed the failure of these major genes in-field due to rapidly evolving pathogen populations (Hovmøller et al. 2016; Ali et al. 2017; Schulthess et al. 2022a). Therefore, mining PGRs for mechanistically diverse disease resistance genes is a high priority to enhance durability. Resistance expressed as a quantitative trait (QR) is often non-race-specific and, based on the evidence of recent gene cloning studies, involves mechanistically diverse modes of action (Milne et al. 2019, 2024; Zhang et al. 2024). QR is challenging to characterize as it is often expressed due to the action of multiple loci of low phenotypic effect (Niks et al. 2015; Dracatos et al. 2023a). Phenotyping QR visually is inherently limited by operator bias, low phenotypic resolution, and is further complicated by genotype × environment (*G* × *E*) interactions when performed in the field. To overcome these limitations and increase the detection frequency of QR in GWAS and further biparental mapping studies, accurate and unbiased image-based phenotyping is required.

In this review, we highlight image-based foliar phenomics approaches that build on traditional resistance phenotyping methods that aim to quantitatively characterize cereal host–pathogen interactions at the macroscopic and microscopic scales to specifically target the phenotypic response of durable QR genes. Key to these approaches is the use of high-throughput image acquisition technology and image analysis using conventional and artificial intelligence (AI) approaches. We also provide an initial survey of the current wheat and barley genetic and genomic resources to help guide targeted GWAS screens for QR in PGRs including core collections and further characterize cereal foliar disease resistance traits.

## Cereal host genetic and genomic resources

Disease resistance research in cereal species will continue to benefit from well-advanced host genetic resources. The vast scale of genebank PGRs poses a significant logistical challenge in prioritizing specific accessions for genotyping and phenotypic screening. Genebank genotyping efforts have generated permanent data resources that link phenotypic variation to genomic diversity to help inform the selection process and to identify gaps or redundancy in collections. Approximately 80,000 wheat accessions from the International Maize and Wheat Improvement Centre (CIMMYT) and the International Centre for Agricultural Research in the Dry Areas (ICARDA) have been genotyped using Diversity Array Technology (DArTseq™) (Sansaloni et al. 2020). The German Federal ex situ genebank for Agricultural and Horticultural Crop species, held by the Leibniz Institute of Plant Genetics and Crop Plant Research (IPK) in Gatersleben (Germany), performed genotyping-by-sequencing (GBS) on 8070 winter wheat accessions and 7240 spring wheat accessions with an additional 768 accessions genotyped using whole-genome sequencing (WGS) data (Schulthess et al. 2022a, unpublished). Previous GBS characterization of 21,405 barley accessions by Milner et al. (2019) paved the way to identify representative genetically and phenotypically diverse genotypes for chromosome-scale reference genome sequencing and assembly as part of the barley pangenomes analysis (v1 and v2) (Jayakodi et al. 2020, 2024). In Australia, the imputation-enabled Illumina Infinium Wheat Barley 40 K SNP array (Keeble-Gagnère et al. 2021; Genebank 2024b, a) was used to genotype 12,606 hexaploid wheat accessions and 13,989 barley accessions from the Australian Grains Genebank (AGG) as part of an initiative to enhance the utility of PGRs for trait identification (Keeble-Gagnère et al. 2021; Genebank 2024b, a). The web-based data browser *Pretzel* addresses the need for an easy-to-use integrated interface for breeders and researchers to visualize and interrogate the 40 K SNP data against recently developed pan genomic resources and historical marker datasets (Keeble-Gagnère et al. 2019). For example, the physical and genetic positions of the 40 K SNPs can be compared to other historical marker datasets such as simple sequence repeats (SSRs) and DArT markers, trait-specific KASP markers, SNPs from different genotyping arrays, QTLs, annotated genes, and syntenic positions from other crops or model species (Bayer et al. 2017; Keeble-Gagnère et al. 2021).

Tremendous progress toward cereal pangenomics has advanced our understanding of important agronomic traits, and this has largely been enabled through long-read sequencing technologies (Montenegro et al. 2017; Jayakodi et al. 2020, 2024; Walkowiak et al. 2020). Historically, genetic research for crop improvement has over-relied on a single reference genome (i.e., barley cv. Morex) that can be problematic when the genome architecture of wild and diverse accessions carrying the allele of interest deviates significantly from the reference. In the Triticeae tribe, complex and/or polyploid genomes with low rates of recombination can be a further barrier (Hatta et al. 2019). The term pangenome can be described as a species-wide catalog of genic presence absence variations (PAVs) from multiple individuals of the same species. Genes cataloged in the pangenome can be classified based on their status across individuals; genes present in all individuals belong to the “core genome,” whereas genes present only in certain individuals are part of the “accessory/variable genome” sometimes referred to as the “dispensable genome” (Hossain et al. 2021). It is critical for the modern breeder to know the status of PAVs and chromosomal inversions in their breeding lines as such variants could unnecessarily complicate breeding efficacy. Knowledge of these variants is particularly important for disease resistance research, where resistance genes often evolve via diversifying selection and duplication events (Lee and Chae 2020). An important example of this is the *Mildew resistance locus a (Mla)* in barley, a complex locus implicated in resistance toward several pathogens (Bettgenhaeuser et al. 2021). The *Mla* locus contains three known nucleotide-binding leucine-rich repeat (NLR) encoding resistance gene families (*RGH1*, *RGH2,* and *RGH3*) and exhibits extensive variation in terms of structural rearrangements, gene copy number, and allelic diversity. The pangenome (v2) revealed a 40 kb region of the *Mla* locus is repeated four times consecutively in the barley cultivar RGT Planet, but is completely absent in 62 accessions (Jayakodi et al. 2024). The exact phenotypic effects of these genome variants on resistance toward barley powdery mildew (BPM, caused by *Blumeria graminis* f. sp. *hordei*) and other diseases remain to be determined and highlight the importance of considering the genetic background of breeding germplasm.

Pangenome datasets now allow the researcher to select a chromosome-scale reference genome closely related to the SNP profile of a resistance donor and have already enhanced gene cloning for rust resistance genes including *Lr14a* (wheat), *Rph3* and *Rph7* (barley) (Kolodziej et al. 2021; Dinh et al. 2022; Chen et al. 2023). Equipped with these genetic and genomic resources, the cereal breeding community is well positioned to greatly accelerate genetic gain of important agronomic traits including plant architecture, nutrient release, trichome development, malting quality, bread quality, and disease resistance (Jayakodi et al. 2020, 2024). These high-resolution genomic resources enable breeders and researchers to delve deeper into the complex genetic architecture of QR. To fully utilize these genomic resources, further image-based phenotyping methodologies are required to build upon visual assessment of disease resistance and characterize this complex trait at high resolution.

## QR for durable resistance toward cereal foliar pathogens

The phenotypic outcomes of disease resistance can be broadly classified as qualitative or quantitative (Stein and Muehlbauer 2018). Qualitative resistance (sometimes referred to as seedling or all-stage resistance) results in near or complete immunity against a single or group of pathogen races, that is, easy to phenotype based on a binary classification as either resistant (*R*) or susceptible (*S*). Qualitative resistance is often associated with canonical classes of immune receptors, containing nucleotide-binding leucine-rich repeat (NLR) or receptor-like kinase (RLK) domains, involved in gene-for-gene interactions with pathogen effectors, yet non-canonical race-specific genes are also known (Jones and Dangl 2006; Dracatos et al. 2019; Chen et al. 2021, 2023; Dinh et al. 2022). More recently, kinase fusion proteins (KFPs) have been implicated in qualitative resistance with the recent cloning of the wheat powdery mildew resistance gene *Pm13* (He et al. 2024). Qualitative resistance is usually expressed due to Mendelian inheritance of a single main locus containing a major *R*-gene that contributes most of the phenotypic variation in QTL mapping studies (Zhang et al. 2013; Stein and Muehlbauer 2018). Major *R*-genes can exert extreme selective pressure on local pathogen populations for effector mutations capable of evading effector triggered immunity (ETI), and in some instances, major *R*-genes have completely failed in the field. Recent pandemics of wheat yellow rust (WYR, causal agent *Puccinia striiformis* f. sp. *tritici*) broke down several *Yr* resistance genes (*Yr9* and *Yr27* in Africa, Australia, and Asia; *Yr17* and *Yr32* in Europe) used in modern wheat varieties (McDonald and Linde 2002; Hovmøller et al. 2016; Ali et al. 2017; Schulthess et al. 2022b).

QR which is also known as Partial Resistance (PR) or Adult Plant Resistance (APR) when assessed in the field, is observed as a reduction, but not elimination of the frequency of disease symptoms, has been well characterized in leaf rust of barley (BLR, causal agent *Puccinia hordei*) (Qi et al. 1998; Hickey et al. 2011; Singh et al. 2015; Niks et al. 2015; Ziems et al. 2017). The expression of QR can extend the latent period (the time from inoculation to visible disease symptom appearance) and reduce the growth rate (disease symptom development over time), which are characteristics of slow-rusting genes. For example, the expression of the cloned barley gene *Rphq2* has been shown to manifest as a non-hypersensitive prolongation of the latent period ranging from 10 to 20% (Wang et al. 2019). The expression of the wheat slow-rusting genes *Lr34*, *Lr46*, and *Lr67* has a major role in delaying the onset of rust development in the field, reducing the onset of epidemics and potential yield loss (Martínez et al. 2001; Singh et al. 2007; Zelba et al. 2024). *Lr34* has been shown to extend the latent period by 24 h and reduce the area under the disease progress curve (AUDPC) by 77% (Singh et al. 2007).

QR is often attributed to epistatic or additive effects of multiple quantitatively inherited alleles that contribute small proportions of phenotypic variance in QTL mapping studies, and is hypothesized to be more durable due to a lack of race-specificity (McIntosh 1992; Navabi et al. 2005; Boyd 2005; Lagudah et al. 2006; Hickey et al. 2011; Niks et al. 2015; Janáková et al. 2019; Dracatos et al. 2023a). However, some authors caution that there is a substantial lack of experimental evidence to validate the durability of QR, and that durability is highly variable across different pathosystems (Niks et al. 2015; Cowger and Brown 2019; Zelba et al. 2024). Using terms such as APR to make assumptions about QR heritability and race-specificity can cause confusion. The known wheat QR genes *Lr34/Yr18/Sr57/Pm38*, *Lr67/Yr46/Sr55/Pm46,* and *Rphq4/Rph20* are inherited in simple Mendelian fashion as a single locus, contrary to the notion that QR is expressed due to inheritance of several minor QTLs (Krattinger et al. 2009; Niks et al. 2015; Moore et al. 2015). Furthermore, race-specific APR genes are known (Park and McIntosh 1994). Therefore, in this review, we define QR as a partial reduction in disease symptoms effective against a pathogen species at the seedling and/or adult stage, expressed due to inheritance of either one main QTL and/or several minor QTL.

Resistance breeding focusing on QR offers several advantages over major *R-*gene mediated resistance. In principle, “pyramiding” or “stacking” of QR alleles in cereals can lead to durable and complete resistance against a pathogen species (Dracatos et al. 2023b). This concept has been trialed in cereals with the  multi-gene cassette referred to as the “Big Five”, comprised of *Lr67/Yr46/Sr55/Pm46* and the all-stage stem rust *R*-genes *Sr22*, *Sr35, Sr45,* and *Sr50,* which have been successfully transferred to rust-susceptible bread wheat via *Agrobacterium*-mediated transformation as a single locus, conferring resistance toward highly virulent isolates of *P. graminis* f. sp. *tritici* (*Pgt*) (Luo et al. 2021). Pleiotropic resistance genes are special class of genes effective against multiple pathogens and are of great value to breeders as they reduce selection complexity during breeding. Three QR genes identified in hexaploid bread wheat confer pleiotropic resistance toward wheat leaf rust, WYR, and powdery mildew (*Lr34/Yr18/Sr57/Pm38*, *Lr46/Yr29/Sr58/Pm39,* and *Lr67/Yr46/Sr55/Pm46*), with both *Lr34* and *Lr67* cloned (Krattinger et al. 2009; Moore et al. 2015; Milne et al. 2024)*.* These advanced resistance gene cloning efforts in cereals have been supported by the keen expert eye visually phenotyping disease resistance.

Attempts have been made to map disease resistance QTLs that control the latency period. For example, the mapping and cloning of the barley gene *Rphq2* (sourced from cultivated barley) was supported by uniform inoculation using a settling tower and visually assessing the latent period (defined as the time period for 50% of pustules to rupture the epidermis) (Qi et al. 1998; Wang et al. 2019). Due to a lack of enabling technologies for high-throughput imaging, targeting growth rates as a specific feature of QR using controlled conditions has remained elusive.

To increase the detection frequency and subsequent characterization of QR in cereals, image-based phenotyping technologies are required to build upon visual assessment and target-specific resistance traits that are controlled by mechanistically diverse genes. Opportunities exist with new technologies to model disease lesion growth rates in large populations as a specific phenotypic trait with high resolution. These methods eliminate operator bias and overcome the limitations of disease pressure varying between years, and other environmental factors. Further developments in microscopy and image analysis aim to uncover mechanistic diversity of resistance during cryptic and early stages of host–pathogen interactions.

## Image-based approaches for phenotyping foliar disease resistance

The wide range of foliar disease symptoms observable by the human eye are used to distinguish resistant and susceptible varieties with the aid of specific rating scales. From the early days of the Cobb wheat rust severity scale, the expert human eye has been guided by visual scales for over a century to phenotype foliar disease resistance by using either nominal, ordinal, or ratio-based scales to score the percentage leaf area covered by disease, the size of individual lesions/pustules, the absence of chlorosis, and hypersensitive cell death (HR) responses (Cobb 1894; Bock et al. 2020). For example, net blotch of barley (causal agent *Pyrenophora teres,* a necrotroph) is scored based on the severity of chlorotic and necrotic symptoms on an increasing scale ranging from 1 to 9 (Tekauz 1985). In cereal rusts, this scale can be even narrower depending on the scale used, ranging from 1 to 4 (Stakman et al. 1962). Breeders and pathologists often record additional comments describing specific features of the phenotypic response associated with specific resistance genes, including necrosis or chlorosis. A major limitation is that in-field phenotyping of disease resistance can only be performed once per season and is subject to environmental and pathogen variability that can mask the effects of QR, especially under high disease pressure (Dracatos et al. 2023b). To avoid these complications and provide the flexibility of disease phenotyping throughout the year, glasshouse seedling assays and detached leaf assays (DLAs) are widely used to complement field-based screening (El-Mor et al. 2018).

Despite the success of resistance breeding and cloning efforts using the human eye, there are well-recognized limitations of visual scoring methodologies that have been extensively investigated in the previous reviews. It is well known that error can be introduced due to rater variation, overestimation bias (e.g., the tendency to overestimate infection area with an increasing number of lesions), bias toward preferred rating values called “knots,” and the environment of the rater (heat, noise, exhaustion, time allocation to score, etc.) (Nita et al. 2003; Sun et al. 2014; Bock et al. 2020, 2022, 2024). Furthermore, it has been proven that the assumptions of the Horsfall–Barratt (HB) scale (that human observers can make better predictions based on logarithmic visual scale intervals) are false (Sun et al. 2014; Bock et al. 2024). Best practice for visual assessment has been well established to minimize error: (i) selection of an appropriate scale for the pathosystem, the requirements of the experiment and resource availability, (ii) providing detailed instructions to the raters in-field and in the glasshouse, (iii) testing and training of raters, (iv) raters must be experienced, (v) where possible use standard area diagrams (SADs) depicting color images of the disease with known percentage infection areas as a visual guide, and (vi) minimize the number of raters in a given experiment to control for rater error, or when multiple raters are involved, they should be assigned randomly across the field plots or the glasshouse (Bock et al. 2020, 2022, 2024). To build upon visual assessment and overcome these limitations, image-based phenotyping aims to detect the subtle phenotypic variation of specific disease symptoms contributed by QR to better understand this complex trait in cereal germplasm, and to further improve mapping of QR genes that could otherwise be masked by the effects of major *R*-genes or confounded by complex (*G* × *E*) interactions.

## Macrophenotyping builds upon visual assessment of foliar disease

Image-based methods to quantify disease are far from novel and have sought to overcome the limitations of visual assessment and improve accuracy, precision, and reproducibility to ultimately increase mapping resolution (Belcher et al. 2012; Xie et al. 2012; Stewart and McDonald 2014; Mutka and Bart 2015; Stewart et al. 2016; Mutka et al. 2016; Divilov et al. 2017; Karisto et al. 2018; Mahlein et al. 2019; Lück et al. 2020, 2025; Hinterberger et al. 2022). Image-based phenotyping involves two key sequential steps: image acquisition and image analysis. Images are stored as multi-dimensional arrays of pixels with a defined height, width, and a third dimension that corresponds to the number of color channels (Szeliski 2022). For example, an RGB image has three color channels: red, green, and blue.

The majority of image-based methods are concerned with assessing disease severity in greenhouses or in the field to inform agronomic management decisions, as opposed to phenotyping disease resistance for crop improvement, and are reliant on various sensors attached to different platforms, including handheld devices, rovers, unmanned aerial vehicles, or satellites (Singh et al. 2016; Kuska et al. 2022). These techniques rely on non-invasive remote sensing that use optical sensors suited for RGB (Hernández-Rabadán et al. 2014), multi or hyperspectral (Bauer et al. 2011; Wahabzada et al. 2015), thermal (Calderón et al. 2013), and chlorophyll fluorescence imaging (Rousseau et al. 2013) as well as 3D image data acquisition (Paulus et al. 2014). Others have attempted to go beyond image-based methods, for example, Koc et al. 2022 employed a spectroradiometer combined with machine learning (ML) models, e.g. random forests (RFs), to predict the presence of yellow rust in wheat using plant senescence reflectance and greenness indices, achieving moderate correlations (*r* = 0.50 – 0.61) between predicted and observed scores.

Image-based approaches to characterize disease resistance have been deployed for cereal species, yet these techniques have not been widely adopted and lack standardization. The general aim of macroscopic characterization has been to segment pixels of healthy versus diseased tissues to derive the percentage of diseased leaf area (Jackson et al. 2007; Stewart and McDonald 2014; El Jarroudi et al. 2015; Stewart et al. 2016; Karisto et al. 2018; Lück et al. 2020; Mathieu et al. 2024; Liu et al. 2024; Anderegg et al. 2024). More sophisticated analysis using AI object detection might aim to recognize specific microscopic features such as fungal pre- and post-penetration structures (for example, sporulation status) (Zelba et al. 2024; Lück et al. 2025). Image-based methods have previously been used to assess QR in-field at the adult stage in a panel of elite winter wheats and advanced recombinant inbred lines (RIL) challenged by *Septoria tritici* blotch (STB, caused by *Zymoseptoria tritici*) (Stewart and McDonald 2014; El Jarroudi et al. 2015; Stewart et al. 2016; Karisto et al. 2018; Mathieu et al. 2024; Anderegg et al. 2024). Images were acquired with RGB cameras and flatbed scanners, and image analysis was performed using ImageJ and ASSESS v. 2.0 software (Image Analysis Software for Plant Disease Quantification; Lamari 2002) to quantify the percentage infection area, the number of lesions, and the number of pycnidia. Phenotypic values derived from the images were significantly correlated to measurements obtained via human visual scoring and showed an improvement upon precision and accuracy, though this method was highly labor intensive taking 360 h for one person to collect and process 22,000 leaves (Karisto et al. 2018). Interestingly, Karisto et al. (2018) further hypothesized that lesions and pycnidia formation were controlled by different genes involved in virulence and reproduction, respectively, and measuring these as distinct phenotypes could be used to map different resistance genes in wheat. El Jarroudi et al. (2015) also utilized ASSESS v. 2.0 software to quantify STB disease severity, achieving high concordance (*ρ*_*c*_ = 0.92–0.99, where *ρ*_*c*_ is a measure of agreement) between predicted and actual severity ratings in field-grown wheat.

The smartphone-based application “StripeRust-Pocket” developed by Liu et al. (2024) aimed to overcome labor-intensive sample collection and uses deep learning (DL)-supported image analysis to segment and quantify pustule area of WYR images taken using a smartphone acquired under natural lighting conditions, and achieved a mean intersection-over-union (MIoU) of 86.08% for lesion segmentation. MIoU is a measure of how well a predicted segmentation result aligns with ground truth data with a known percentage infection area. Studies on other cereal pathosystems such as oat crown rust (OCR, caused by *Puccinia coronata* f. sp. *avenae*) have also leveraged quantitative image analysis—Jackson et al. (2007) utilized flatbed scanners in combination with ASSESS software to measure pustule area in RIL populations under both glasshouse and field conditions. Their findings highlighted the potential of image analysis to increase mapping accuracy of an unknown major gene, where 52% of phenotypic variation was explained using phenotypic measurements obtained from digital images compared to 41% by human visual scoring.

At the seedling stage, similar image-based methods have been used to phenotype STB symptoms (Stewart and McDonald 2014; Mathieu et al. 2024). Stewart and McDonald (2014) used RGB cameras and ImageJ to quantify STB lesion area and pycnidia metrics in wheat grown in glasshouses, demonstrating higher accuracy and precision (*C*_*b*_ = 0.97, *r* = 0.97) compared to traditional visual assessments (*C*_*b*_ = 0.83, *r* = 0.80), where *C*_*b*_ is a bias correction factor and gives a measure of accuracy, and *r* is Pearson’s correlation coefficient. More recently, the open source “SeptoSympto” software was developed in Python, using U-Net and You Only Look Once (YOLO) model DL architectures to segment and quantify STB necrosis and pycnidia on wheat leaves at the third leaf stage (Mathieu et al. 2024).

**Fig. 1:**
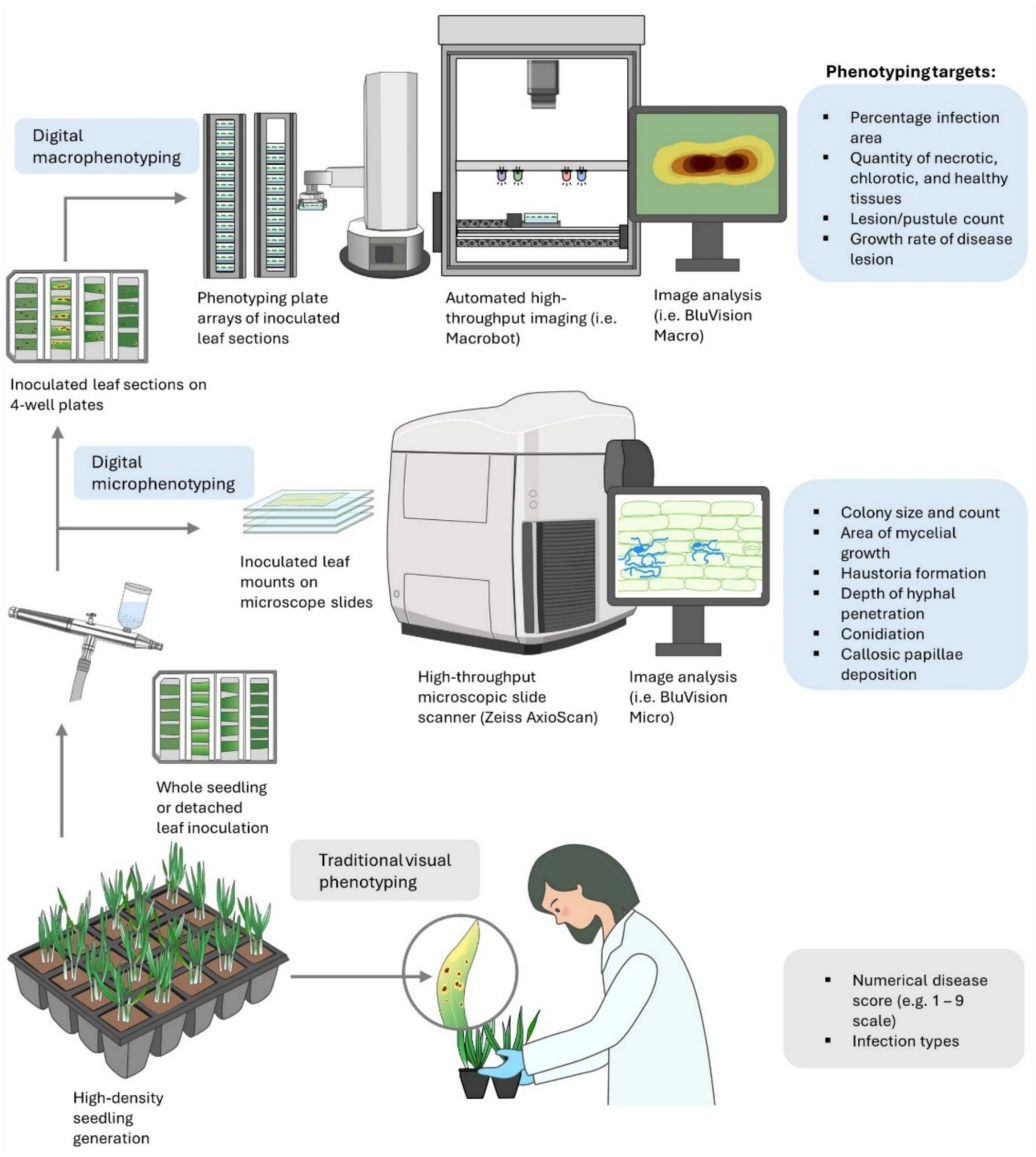
Comparison of traditional visual phenotyping of disease resistance to image-based macro- and micro-phenotyping. Phenotyping using the human eye is limited to numerical scores and infection types, whereas digital phenotyping can quantitatively measure multiple aspects of the host–pathogen interaction resistance macro- and microscopically. Image-based phenotyping workflows, such as those used at the IPK (Germany) and most recently deployed at La Trobe University (Australia), involve an initial controlled inoculation either on detached leaves or as whole seedlings that are mounted onto four wells (containing water agar, silver nitrate, and a senescence inhibitor) to image live leaves during the progression of disease development. A robotic arm automatically loads plates from magazines with a capacity sufficient to hold a whole experiment. Images are acquired with a monochrome digital camera on a stage selectively illuminated by LED lights with peak wavelengths 365 nm (UV), 470 nm (blue), 530 nm (green), and 625 nm (red). The standardized images are analyzed by dedicated software modules published on open-source GitHub repositories that can be modified for specific pathogens (Lück et al. 2020). Digital microphenotyping workflows such as “BluVision Micro” rely upon commercially available slide scanners common in medical pathology to reduce laborious sample handling and acquisition such as the Zeiss® Axioscan (Carl Zeiss Group). These microscopic workflows rely upon specific staining steps to facilitate ease of visualization and subsequent image analysis, and the choice of stain is highly dependent upon the pathosystem under investigation

One notable image-based approach developed at the Leibniz Institute of Plant Genetics and Crop Plant Research aimed to specifically target QR in vast cereal germplasm screens using uniform, controlled inoculation conditions necessary to detect QR that could otherwise be masked in the field or greenhouse due to variable disease pressure (Fig. 1) (Lück et al. 2020, 2025). The group aimed to characterize macroscopic and microscopic resistance phenotypes toward wheat powdery mildew (WPM, *Blumeria graminis* f. sp. *tritici*) using largely automated sample handling, and image analysis supported by conventional methods and/or AI. The Macrobot (Compolytics®, Barleben, Germany) imaging hardware platform is central to the BluVision foliar phenomics pipeline used for screening germplasm in DLAs (Lück et al. 2020). Similar hardware designs for pre-symptomatic disease detection using hyperspectral imaging have been described, but were not optimized for high-throughput DLAs of cereal crops (Zhu et al. 2017).

The Macrobot uses a monochrome camera (Macrobot Next Gen is equipped with an Atlas 1UV 8.1 MP 16-bit monochrome camera) to image detached leaves in plates illuminated with narrow-band wavelength light-emitting diodes (LEDs, usually with peak wavelengths corresponding to RGB, plus additional channels in the ultraviolet, UV, or near infrared, NIR). Monochrome cameras are widely used in multispectral imaging systems due to their ability to capture accurate, band-specific reflectance values without interpolation artifacts associated with Bayer-pattern color sensors (Forsey et al. 2016; Hijazi et al. 2022). Bayer sensors, typically found in consumer color cameras, allocate subsets of pixels to red, green, or blue using filters and reconstruct a full-color image via demosaicing. Monochrome sensors utilize the full pixel array to capture light at each LED target wavelength, yielding true spectral reflectance values at every pixel. This eliminates color blending and moiré artifacts, and provides increased spatial resolution and photon sensitivity, as there are no absorptive microlens arrays or color filters blocking specific wavelengths (Forsey et al. 2016). The use of narrow-band LEDs produces clean, cross-talk free reflectance data with high signal-to-noise ratios, that can be extended to non-visible wavelengths such as NIR and UV without modifying the sensor hardware. Furthermore, using monochrome cameras simplifies image processing, as each channel corresponds to a known wavelength and requires only per-band radiometric calibration, without the need for complex demosaicing pipelines that can degrade spatial and spectral fidelity (Wang et al. 2021b).

The typical Macrobot plate layout has four wells that correspond to a different genotype, with capacity for eight biological replicates per lane. Following inoculation, DLA plates are maintained in growth chambers under controlled temperature, humidity, and lighting conditions that are biologically relevant to the host–pathogen system being studied. A key aspect that differentiates the Macrobot from other approaches is the ability to derive the latency period and growth rates of disease within the same biological replicate to specifically phenotype these features of QR. BluVision Macro was used to screen 8316 genotypes at the seedling stage from the winter wheat collection of the German Federal ex situ Gene Bank of Agricultural and Horticultural Crop Species for resistance toward WPM (Hinterberger et al. 2022). The quantitative phenotypic data obtained with the Macrobot supported a GWAS that revealed 51 significant MTAs, and when compared against the SNP profiles of 171 elite European winter wheat varieties, 11 of these MTAs were found to be in chromosomal regions not previously associated with powdery mildew resistance when anchored to the reference genome Chinese Spring (IWGSC RefSeq v1.0) (Hinterberger et al. 2022).

Attempts have been made to assess the gain in QTL mapping resolution using image-based approaches to phenotype foliar disease resistance, although this important area has been largely understudied in cereals. Jackson et al. (2007) investigated this in their mapping population of F_6:9_ recombinant inbred lines (RILs) when attempting to map an unknown major gene and found differences in the mean marker position using visual estimates versus image analysis; in the greenhouse, this difference was 2.3 cM, and in the field, this was 1.0 cM. Future image-based approaches to support resistance gene cloning should aim to incorporate detailed validation studies using pathosystems with known cloned genes (such as the cereal rusts) to assess the gain in mapping resolution when using different phenotyping methodologies.

## Microphenotyping provides deep mechanistic understanding of host–pathogen interactions

As previously discussed, the macroscopic assessment of disease resistance in plants has been improved with the advent of automated image-based methods under controlled conditions. These high-throughput systems show promise in uncovering mechanistically novel plant resistance genes (Hinterberger et al. 2022). While this information is valuable, macrophenotyping overlooks resistance mechanisms active at the early stages of infection. These early and often asymptomatic or cryptic responses are subtle yet crucial, as they are likely controlled by more durable QR genes which provide long-term non-race-specific protection from fungal pathogens (Dracatos et al. 2023a).

Microscopy has long been instrumental in exploring plant–pathogen interactions and has revealed the cryptic aspects of fungal pathogenesis and plant resistance mechanisms (Rajarammohan 2021; Czymmek et al. 2023). Infected plant tissue is commonly stained with fungal cell wall specific dyes such as lactophenol blue or fluorescent dyes including wheat germ agglutinin conjugates or Uvitex 2B to visualize pathogenic fungi *in planta* (Lightfoot and Able 2010; Porras et al. 2022; Nelson et al. 2024). These staining methods have provided valuable insights into fungal biology, facilitating the distinction between biotrophic, necrotrophic, and hemibiotrophic lifestyles (Gan et al. 2013; Chowdhury et al. 2014). Additionally, comparative analyses between resistant, susceptible, and non-host plant genotypes can be performed (Dracatos et al. 2014). This can be further extended into the assessment of fungal colony characteristics such as hyphal branching patterns, hyphal expansion, colony density, and biomass accumulation (Lightfoot and Able 2010; Nelson et al. 2024; Lück et al. 2025).

Microscopy has highlighted that resistance can occur at all stages of infection. Pre-invasion defenses include structural and chemical characteristics of the leaf surface that hinder pathogen establishment. Physical barriers such as trichomes may influence the number of spores that successfully land on the leaf surface, and the presence of a waxy cuticle can affect spore adhesion and germination (Nielsen et al. 2000; Niks and Rubiales 2002). Stomatal traits, including density, morphology, and the ability to rapidly close in response to pathogen detection, also have a crucial role pathogen defense (Schauffler et al. 2022; Meddya et al. 2023). For example, maize genotypes resistant to *Bipolaris maydis* were found to have fewer stomata than susceptible genotypes and a higher deposition of surrounding wax (Manjunatha et al. 2019). Similarly, in chickpea, smaller and fewer stomata were observed in a genotype resistant to *Botrytis cinerea* (Thakur et al. 2023).

Post-penetration mechanisms include those that inhibit haustoria formation (Ayliffe et al. 2011; Dracatos et al. 2014; Mapuranga et al. 2022), and later-stage barriers that restrict pathogen expansion, sporulation, and secondary spread (Schnippenkoetter et al. 2017; Porras et al. 2022; Sharma et al. 2024). Pre-haustorial resistance mechanisms involve cell wall reinforcements such as callose deposition which restrict pathogen entry and slow its spread (Wang et al. 2021a). Aniline blue staining has revealed callose accumulation at the sites of fungal penetration and surrounding haustorial complexes, forming specialized projections known as papillae (Stolzenburg et al. 1984; Ayliffe et al. 2011; Dracatos et al. 2014; Wang et al. 2021a). In barley, *Ror1* was required for *mlo*-derived callose deposition resistance to BPM (Aghnoum et al. 2010; Acevedo‐Garcia et al. 2022). Similarly, QTL and MTA analysis complemented with callose staining identified *Rpga2* as another gene likely contributing to non-host resistance to *P. graminis* f. sp. *avenae* (Dracatos et al. 2014).

Post-haustorial resistance includes the activation of ETI resulting in programmed cell death (PCD), which has evolved in defense against biotrophic fungal pathogens but manipulated to benefit necrotrophic fungal pathogens (Liu et al. 2024). The activation of PCD has been extensively studied in the *Parastagonospora nodorum*-wheat interaction, induction of which has been linked to the putative effector SnTox1 (Kariyawasam et al. 2022, 2023). Propidium iodide counterstaining has been used to precisely time the activation of PCD, revealing that necrotic pathogens induce cell death once fungal biomass has accumulated (Nelson et al. 2024). Another form of post-haustorial resistance that can be assessed microscopically is the inhibition or reduction of sporulation. The reduction in numbers, size, and the rate of development of uredinia in rust species is a key feature of QR in cereals, as conferred by *Lr34*, *Lr46,* and *Lr67* in wheat (Bettgenhaeuser et al. 2014; Zelba et al. 2024).

Although microscopic analyses allow the differentiation of resistance responses to fungal pathogens and the visualization of fungal structures *in planta*, microscopy is often time consuming, lacks true high-throughput capacity, and is not quantitative. Recent advances in image analysis techniques, aided by AI, have enhanced our ability to quantitatively measure fungal colony growth and phenotypic markers of resistance. For example, Zelba et al. (2024) applied deep learning to assess fungal infection area and uredinia development in a *P. striiformis* f. sp. *tritici*-wheat interaction. Similarly, Nelson et al. (2024) utilized ML to determine the fungal biomass and PCD in *Parastagonospora nodorum* (Pn), *Pyrenophora teres* f. *teres* (Ptt), and *Cercospora beticola* infections in wheat, barley, and sugar beet, respectively.

While these approaches yielded quantitative outcomes, imaging remains labor-intensive which is a challenge for scaling studies to encompass the screening of large germplasm collections. BluVision Micro is an automated microscopy pipeline developed at the IPK (Gatersleben, Germany) to specifically characterize genebank-wide variation of resistance toward wheat and barley powdery mildew (Fig. 1) (snowformatics 2024; Lück et al. 2025). This microscopy approach relies on Coomassie blue staining of epidermal mildew colonies to enable ease of pathogen visualization, image acquisition, and analysis. Key to the success of BluVision Micro is AI-assisted image analysis of whole-slide images acquired using the Zeiss® Axioscan. The output data of the pipeline summarizes the total number of fungal micro colonies, and colony area, number of mildew colonies, and the sporulation status of colonies. The most promising feature of the BluVision microscopy pipeline is the ability to catalog the micro-phenotypes (that may be the result of pre- and post-haustorial resistance mechanisms) of known and uncharacterized resistance genes either present as single genes or in combinations.

## Image analysis: traditional and AI-based approaches

Arguably, the most groundbreaking aspect of image-based resistance phenotyping is automated quantitative image analysis using traditional and AI methods, that enables further dissection of cereal host plant resistance phenotypes. Due to the increasing prevalence of AI in digital agriculture and its utility in “big data” analysis, the next section of this review provides an overview of key concepts in AI to help non-experts become fluent in AI terminology and methods, and to bridge the gap in understanding within multidisciplinary teams (Kuska et al. 2022). Specifically, we focus on the application of AI for the specific tasks of image segmentation and object detection, the AI model training process, AI model validation/transparency, and the leading approaches and algorithms that are currently being used for disease resistance phenotyping in cereal species.

**Fig. 2:**
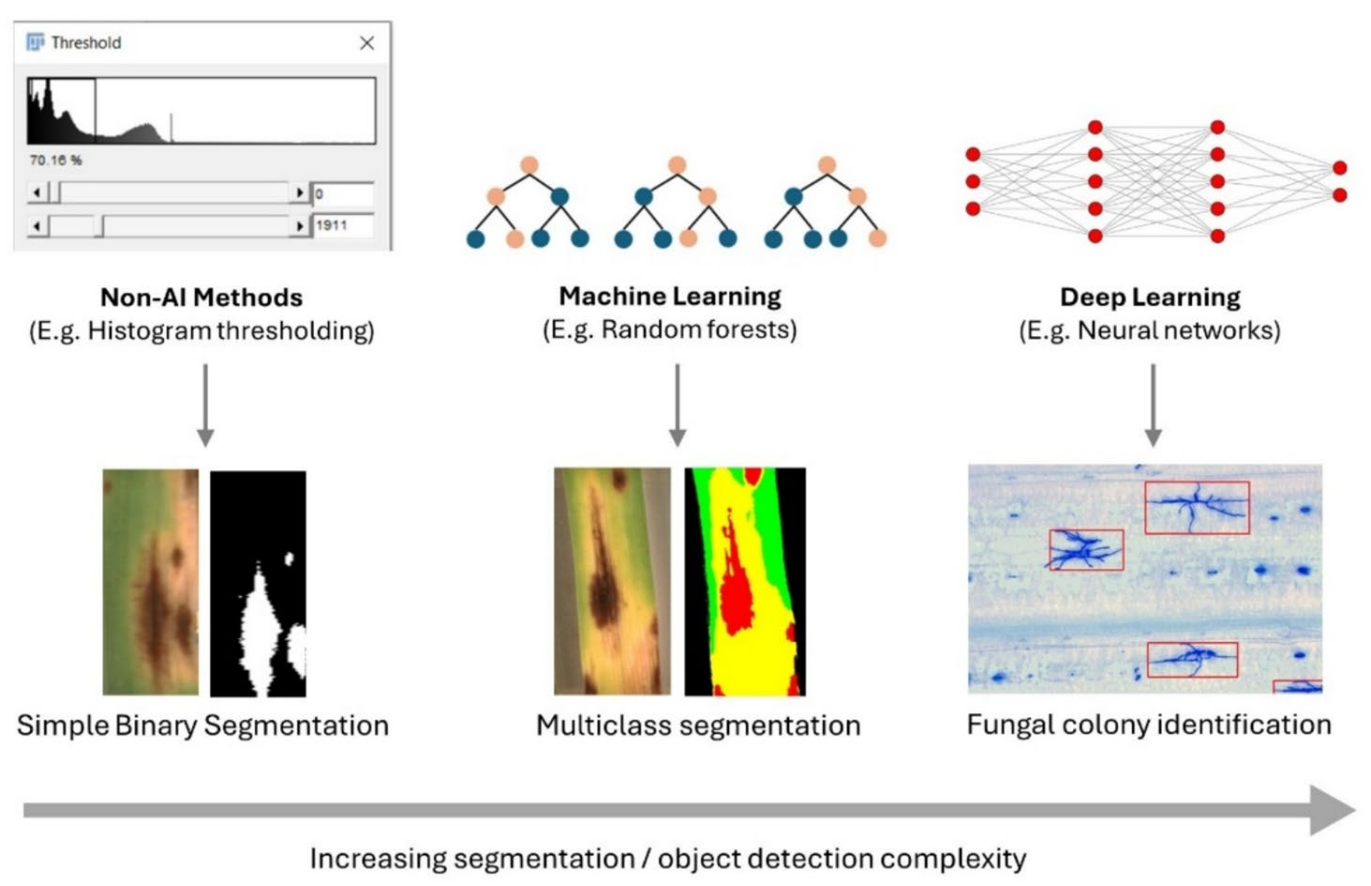
Traditional and AI approaches for image segmentation. Histogram thresholding involves finding a fixed or dynamic pixel intensity value to delineate the diseased versus healthy tissue (Schindelin et al. 2012). In contrast, AI methods learn complex patterns between pixels to achieve segmentation or object recognition tasks

Segmentation is a required step for the quantification of diseased area, and the decision to use traditional or AI image segmentation approaches is largely based on the complexity of the task. Traditional image thresholding techniques are well suited for simple binary segmentation of healthy versus diseased pixels if there is sufficient contrast and bit depth, or ML and DL suited for highly complex segmentation tasks with many classes and recognition of specific objects (Fig. 2 and Table 1) (Arzt et al. 2022; Kirillov et al. 2023; Yu et al. 2023). Traditional image thresholding is not computationally demanding, usually faster in terms of processing time and does not require training but does require manual feature engineering and threshold finding. A case in point, the image analysis of wheat powdery mildew resistance at the seedling stage in the German Federal ex situ Gene Bank winter wheat collection was performed using a non-AI algorithm, that achieved the highest segmentation accuracy compared to ML methods (Lück et al. 2020; Hinterberger et al. 2022). This segmentation algorithm  identified areas of minimal intensity in the image using Minimum Intensity Projection (MIP) maps. The decision to use AI should be carefully considered against the complexity of the segmentation or object detection task.

**Table 1:** Comparison of the relative advantages and limitations between traditional, ML (non-DL), and DL image analysis techniques for segmentation and object detection

Aspect	Traditional image analysis	Machine learning (non-DL)	Deep learning (DL)
Feature extraction	Manual (e.g., color thresholds, and edges) Yu et al. (2023)	Manual + statistical modeling (e.g., shape, color, and texture features) Singh et al. (2016); Rai (2024)	Supervised or automatic (learns features from raw images) Singh et al. (2016); Rai (2024)
Advantages	Simple & interpretable	Can model complex relationships	High accuracy with large training datasets
	Low computational cost Lück et al. (2025)	Works with moderate data	Learns hierarchical features (e.g., high-level salient features and deep features)
Limitations	Poor generalization to complex image datasets	Needs careful feature engineering	Requires large, labeled datasets
	Sensitive to lighting/noise		High computational demand Robertson et al. (2018)
Typical algorithms	Histogram thresholding, edge detection, region growing, color filters Schindelin et al. (2012)	Support vector machine (SVM), RFs, multiple instance learning (MIL) Breiman (2001); Singh et al. (2016)	CNNs, U-Net, ResNet, YOLO, Mask R-CNN Liu et al. (2024); Mathieu et al. (2024)
Data requirement	Low	Moderate	High
Applications	Binary segmentation Schindelin et al. (2012)	Multiclass segmentation Berg et al. (2019); Lück et al. (2020); Arzt et al. (2022); Nelson et al. (2024)	Complex blurred edge segmentation and object detection Rai et al. (2024), Lück et al. (2025)
Ease of use	Easy (ImageJ, OpenCV scripts)	Moderate	Specialized programming knowledge and hardware is required

When more complex segmentation or object detection is required, AI methods are well suited to the task. AI is a broad term referring to techniques enabling a machine to copy or exceed human intelligence (Sheikh et al. 2023). This definition includes classical rule-based systems of AI where knowledge of human experts is translated into code for the purpose of decision-making. Breakthroughs in ML and DL have achieved highly complex segmentation and object detection tasks not possible with conventional algorithms (Krizhevsky et al. 2017; Kirillov et al. 2023; Rai 2024). Non-experts often confuse the terms AI, ML, and DL and use them interchangeably; ML is a subset of AI, and DL is a subset of ML. The output of ML and DL segmentation models is a balance of probabilities that a pixel belongs to a given class (e.g., 60% probability diseased and 40% healthy; therefore, the pixel is assigned to the diseased class). The power of ML and DL models comes from their ability to learn highly-complex abstract patterns and associations between pixels (known as features) that are not always obvious to the human eye (Singh et al. 2016). Although this has been applied relatively recently to disease resistance in cereal crop pathology, ML and DL methods have completely revolutionized medical pathology image analysis (Robertson et al. 2018; Pavicic et al. 2021; Gutiérrez et al. 2024; Lück et al. 2025).

## Training AI segmentation and object detection models

**Fig. 3:**
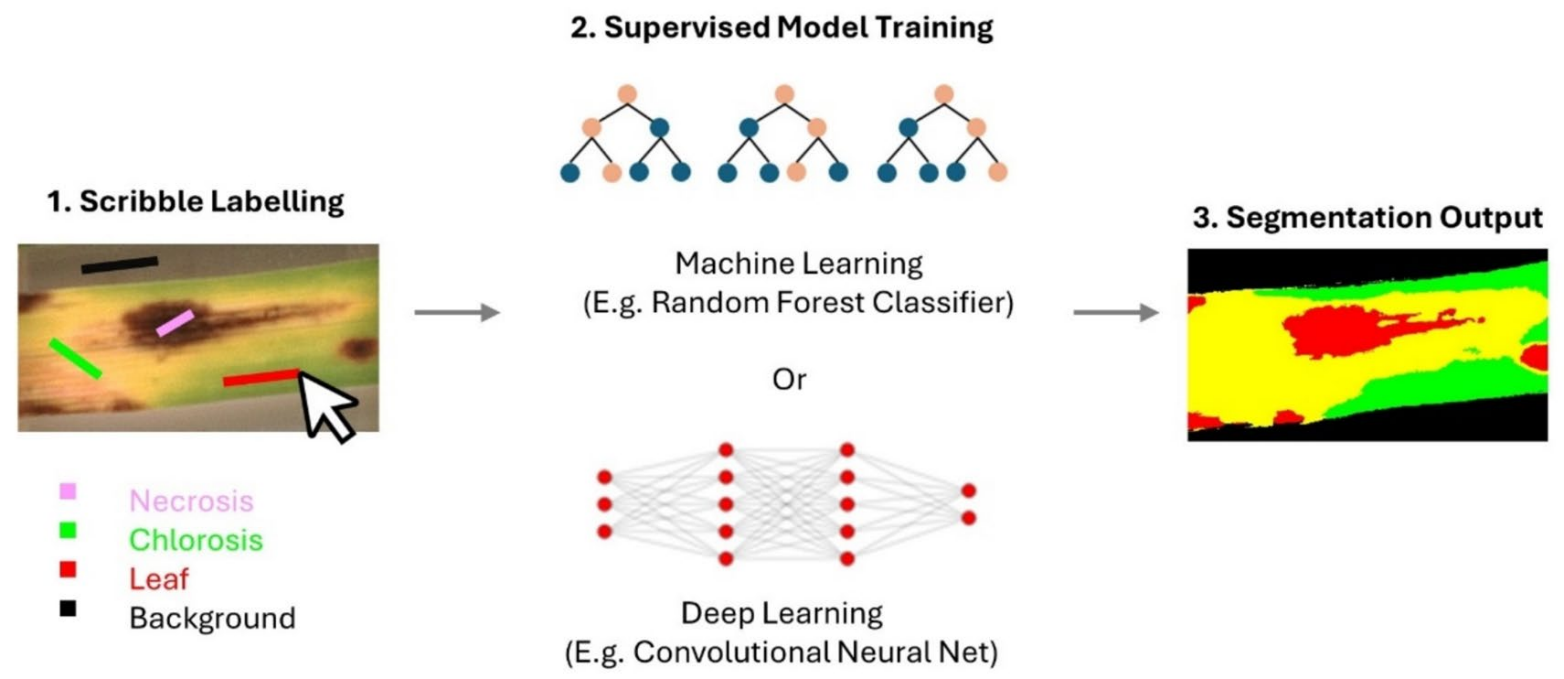
Example of typical labeling process to train AI models. The user manually draws scribble labels to indicate where diseased versus healthy tissues are located. The model is then trained to learn associations between where the labels are located and the underlying pixels. The output segmentation map is then compared against the labels to confirm the accuracy of the model

Supervised learning is a common AI training approach, that requires expert pathologists to manually annotate disease image training data with labels to “show” the model the location of diseased/healthy pixels (or the position of objects) which is known as ground-truth (Robertson et al. 2018; Lueck et al. 2020; Xu et al. 2020; Gutiérrez et al. 2024). Popular open-source labeling software is available including LabKit (as part of FIJI analysis freeware), Ilastik, QuPath, Labelme, and Napari (using specific plugins from Napari Hub) (Bankhead et al. 2017; Berg et al. 2019; Arzt et al. 2022; Chiu et al. 2022; Wada 2025). Labels can be windows/bounding boxes encompassing the diseased area, or alternatively can be drawn using a pen tool to assign a class (e.g., chlorosis, necrosis, and sporulation) to individual pixels (Fig. 3) (Singh et al. 2016; Sager et al. 2021). These tools also have simple ML-assisted functions to automatically label an entire image, which overcomes the tedious task of manually labeling individual pixels in large datasets (Berg et al. 2019; Arzt et al. 2022). The image data and associated labels are then used to train the model, and it is common practice to split datasets into 70% for training, and retain 30% for a validation step that tests that the model is predicting with sufficient accuracy (Singh et al. 2016). There is a trade-off between time and money spent on labeling and the final accuracy of the model; however, training is an iterative process that can be improved as more image data is acquired. Unsupervised learning is a more difficult approach where no labeling is used, and the model must learn to detect the salient features of the image (Singh et al. 2016).

ML and DL models must be trained with sufficient image data to ensure accurate performance prior to deployment. ML techniques, particularly DL, only achieve high accuracy with large volumes of data (Robertson et al. 2018). In some cases, creating a sufficiently large dataset can be challenging. One solution is data augmentation, which involves applying various image transformations to create “new” images from a smaller image dataset (Shorten and Khoshgoftaar 2019; Yang et al. 2023). This includes techniques such as flipping, rotating, cropping, and less often altering color, contrast, adding blur, scaling, and elastic deformation. Processing input images at different magnifications is also a common technique. Covariate shift can be a major problem where the distributions of “live data” (data outside of the training dataset) deviates from the training distribution that can cause the model's performance to deteriorate (Barbedo 2022). For example, an AI model trained on images obtained using one brand of hardware equipment may fail when exposed to images acquired using a different hardware platform (known as “overfitting”) (Robertson et al. 2018). These minimal alterations caused by the image hardware can completely mislead the model, even though the image appears unchanged to humans (Szegedy et al. 2013). Using standardized image acquisition hardware such as the Macrobot® and Zeiss Axioscan® can minimize this problem.

Another option to overcoming the limitations of small datasets is the use of transfer learning, that involves using models trained on large datasets. Transfer leaning was used in the development of “SeptoSympto” for pycnidia detection on wheat leaves, where the pre-trained YOLOv5 architecture was fine-tuned on a database of 240 leaf images (Mathieu et al. 2024). Similarly, Liu et al. (2024) developed “StripeRust-Pocket” based on pre-trained models for leaf segmentation (using MobileNetV2-DeepLabV3 +) and lesion segmentation (using ResNet50-DeepLabV3 +).

**Fig. 4:**
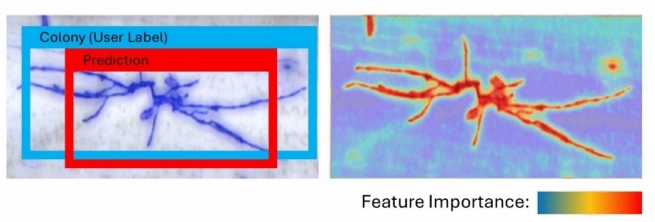
Examples of visualization tools used to validate AI models are recognizing the salient features of the image, and not overfitting on irrelevant parts of the image. The first case compares the user-defined label (the blue bounding box) to the AI prediction (the red bounding box). The second example depicts a class activation map (CAM) or saliency map. CAMs are a heat map that enables the visualization of important pixels used in the model prediction

There is often a lack of transparency as to how DL models make predictions, which can lead to useful results but uncertainty as to how they were obtained. Tools have been developed that help to depict how a model “sees” the image and are important for assessing what parts of the image the model assigns importance, and if the model can detect the salient features at all (Jiang et al. 2021; Lee et al. 2021) (Fig. 4).

## AI machine learning for image segmentation: non-DL algorithms

There are several non-DL machine learning algorithms that have been highly effective at image segmentation tasks. Nelson et al. (2024) used the RF segmentation capability of LabKit and Imaris to segment fluorescence microscopy images of *Ptt* and *Pn* mycelium within leaf tissues of susceptible barley and wheat genotypes, respectively. The segmentation results were used to derive volumes of fungal biomass. Comparative analysis using this RF image analysis approach with resistant genotypes of wheat and barley is yet to be performed, though a reduction in two-dimensional surface area of mycelium has been associated with genotypes carrying *Yr18*, *Yr29,* and *Yr46* (Zelba et al. 2024; Milne et al. 2024). RFs are a widely used algorithm that employs a collection of decision trees (known as an ensemble) on random subsets of data to derive a consensus result, that enhances its robustness against data variations (Fig. 2) (Breiman 2001). The RF algorithm has been used for diseased leaf area segmentation, and also is used for ML-assisted labeling/segmentation in LabKit, Imaris, and Ilastik software (Fig. 3) (Berg et al. 2019; Lück et al. 2020; Arzt et al. 2022; Nelson et al. 2024). RFs break down the decision-making process into a sequence of tests, creating a tree-like structure. Each decision is represented by a node, starting from a single root node. At each node, a test is applied to the input sample, and based on the result, the sample is passed to one of the node's children. This process continues until a leaf node is reached, which decides in which class a pixel belongs. Other commonly used ML algorithms include SVM, nearest neighbors, and MIL (Singh et al. 2016).

## Deep learning for image segmentation and classification

**Fig. 5:**
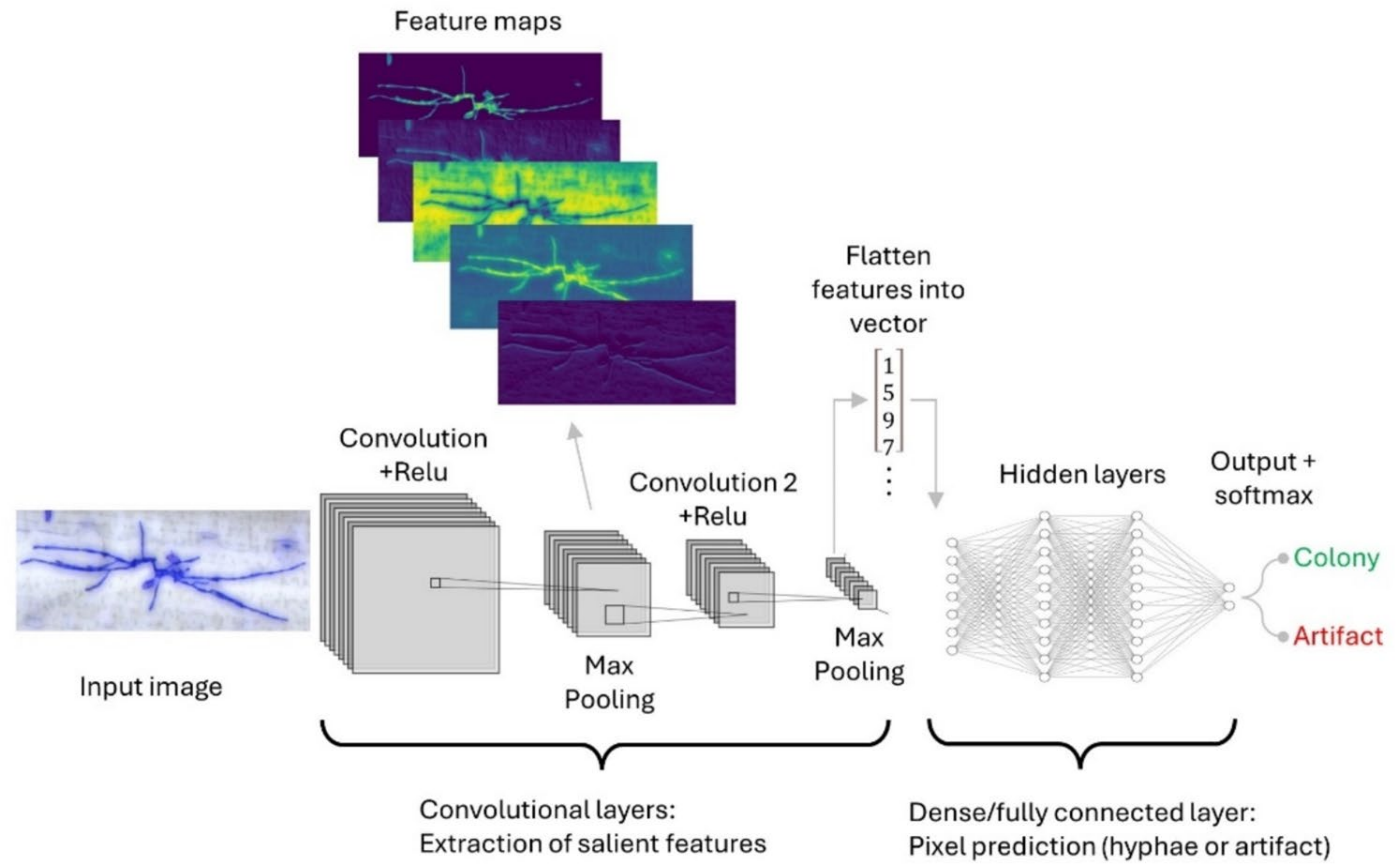
Simplified example of CNN architecture for fungal hyphae detection. Putative images of fungal microcolonies are input to the model as a three-dimensional array known as a tensor, where the height and width are defined in pixels and the depth is represented by the number of color channels. Filters (also known as the kernel) represent small matrix equations with depths equal to the input image. The filters are applied to the image in a process known as convolving where the filter is moved across the input image and the output is a feature map (or activation map). The stride is the number of pixels the filter moves across the input image in one step, where a larger stride results in smaller output feature maps. An activation function such as rectified linear activation unit (ReLU) is usually applied to introduce nonlinearity to the model by converting all negative matrix values to zero. The pooling layer is used to reduce the dimensions of the image as it passes through the convolutional layers, which can help reduce computational complexity and prevent overfitting (Dhillon and Verma 2020; Li et al. 2022). There are two kinds of pooling operation; maximum pooling gives the maximum value within the kernel, whereas average pooling gives the average value of the region covered by the kernel. The pixel prediction (determining fungal colony vs background pixels) is performed by the dense/fully connected layer, and the image must be converted into a one-dimensional array prior to this. The output of the model assigns a class to the image as either colony or background artifact

DL models excel at highly complex segmentation and object detection tasks, and have enabled detailed image analysis specifically targeting resistance mechanisms affecting different stages of infection. One prominent example successfully used DL to segment fluorescence microscopy images of WYR infections in wheat lines carrying functional and non-functional alleles of *Yr18*, *Yr29,* and *Yr46* (Zelba et al. 2024). A U-Net DL model was trained to segment sporulating regions of WYR mycelium to elucidate the microscopic effects of these genes on pathogen growth and reproduction. It was concluded that *Yr18*, *Yr29,* and *Yr46* restrict colonization and pustule formation of WYR, although the effects of *Yr29* were less pronounced. DL models learn from large datasets autonomously by developing an artificial network of neurons (nodes representing mathematical functions, edges representing connections between nodes and the flow of input information) that can identify highly complex nonlinear patterns in data (Fig. 5) (LeCun et al. 2010). There are several types of deep networks highly effective for image analysis; convolutional neural networks (CNNs), autoencoders, recurrent networks, and adversarial networks (LeCun et al. 2010; Salehinejad et al. 2018). Deep belief networks and restricted Boltzmann machines are also commonly used (Hinton 2009; Salakhutdinov and Hinton 2009; Pinaya et al. 2020). More recently, generative adversarial networks (GANs) have gained attention (Huang et al. 2017).

Because of the success of CNNs in many fields such as computer vision and natural language processing, and their potential to lead to breakthroughs in quantitative genetics, we discuss CNNs in detail compared to other models (Dhillon and Verma 2020; Li et al. 2022). The success of CNNs is largely due to their ability to learn high-level features that help the model overcome overfitting problems on non-salient features of the image. The mobile application “StripeRust-Pocket” mentioned earlier uses a two-stage CNN segmentation model to initially segment the outline of whole wheat leaves imaged in-field, which is then processed by the disease segmentation model to extract the lesion area of WYR (Liu et al. 2024). Although this application is primarily designed to inform agronomic management decisions, it has not been used to investigate QR in field in diversity panels or advanced biparental populations. The BluVision Micro pipeline is another prominent example of CNN model usage, aiming to detect microscopic colonies of BPM in a diversity panel of 196 barley accessions (Lück et al. 2025). Phenotypic trait data derived from the CNN model enabled the counting of microscopic BPM colonies at 48 h post-infection was used to support a GWAS that identified significant peaks on chromosomes 3H and 7H (relative to Morex V2). The use of microscopic trait data in GWAS is a relatively unexplored aspect of disease resistance research and holds the possibility of unlocking diverse resistance mechanisms that are distinguishable using the human eye.

CNNs consist of multiple layers, and the three primary layers of a CNN are the convolutional layer, pooling layer, and fully connected layer (also known as the deep layer) (Fig. 5) (LeCun et al. 2010; Tang et al. 2022). Importantly, the convolutional model extracts the salient features of the images, and the classification model makes the final prediction for in object detection or segmentation tasks (Bock et al. 2020). The fully connected or dense layer of a CNN plays a major role in making final predictions (Dhillon and Verma 2020; Li et al. 2022). The fully connected layer receives information from the convolutional layers as a one-dimensional vector (a matrix with width 1 and height *n*). The final output of the fully connected layer is a probability distribution that a pixel belongs to a certain class (for example in a segmentation task a pixel is assigned; 60% diseased and 40% healthy). The probability distribution is then used to construct the segmented image, for subsequent quantitative analysis. In an object detection DL model, the output could be a bounding box prediction encompassing the area of the object (for example, detection of fungal colonies, see Fig. 4).

## Future perspectives

Digital images facilitate easier communication between experts and can be shared for educational purposes or remote collaborations and will form part of modernized passport data, representing an exact likeness of the accessions’ specific disease response under controlled conditions. This will largely depend on open access data sharing policies under collaborative agreements. Image-based phenotyping for foliar disease resistance is not expected to replace conventional in-field and glasshouse assessment by expert pathologists, but provides a quantitative tool to address the accuracy and environmental variability associated with field phenotyping, and detect subtle differences and small effects of QR genes. Detailed characterization of host–pathogen interactions using image-based phenotyping will enable the targeted identification of genes responsible for different aspects of the infection process such as initial penetration, reproduction, and ability to damage the host that will ultimately allow breeders to increase the mechanistic diversity of resistance genes in their breeding lines. These approaches aim to capture as much phenotypic variation possible to improve mapping resolution when specifically targeting QR. The high-throughput automation capacity of these enabling technologies addresses the need to catalog the resistance status of germplasm collections with both accuracy and efficiency at scale. As mentioned, dissecting resistance traits such as increased latency period and reduced growth rates is a novel aspect offered by enabling technology such as the Macrobot and provides a unique opportunity to identify new QR loci in cereal diversity collections.

A largely under-investigated aspect of image-based resistance phenotyping is the improvement in mapping resolution compared to using the human eye, and whether image-based measurements obtained under controlled conditions translate to disease resistance ratings obtained via whole seedling screens in the glasshouse and in-field adult plant observations. It will be an important next step to benchmark these image-based approaches using advanced mapping populations carrying known resistance genes. Although these image-based techniques are primarily concerned with phenotyping QR, it would be interesting to assess the improvement in mapping known major *R*-genes in rust or mildew pathosystems. It is expected that using image-based phenotyping approaches will enhance the ability to map other contributing minor-effect loci contributing to the resistance phenotype.

The available genetic and genomic resource for cereals now enable the breeder or researcher to survey the abundant diversity of genebanks and target representative core collections for QR, among other important traits. It would be interesting to catalog the resistance status of cereal pangenome accessions using multi-pathotyping for further comparative genomics studies to understand the complex genetic architecture of QR. If pangenome germplasm is not immediately accessible to perform these multi-pathotype screens, the researcher may choose to identify the most closely related SNP profiles of genotyped genebank accession to use in proxy for the desired pangenome line, with the caveat that the proxy line does not truly carry the full genetic background of the pangenome line. Importantly, breeders can assess the probability of complicated PAVs in the vicinity of significant MTAs by validating the collinearity of marker positions across diverse pangenome accessions. Another useful approach is to use a significant MTA identified in GWAS to determine how common that SNP resistance haplotype is among pangenome lines, elite cultivars, and other genotyped germplasm (elite breeding material, core collections, etc.), and then assess the stability of the resistance phenotype in different genetic backgrounds. This is critical for determining the reliability of markers as a predictor of resistance, especially considering the complex nature of resistance loci (Bettgenhaeuser et al. 2021).

The core aim of association genetics to investigate disease resistance is to accurately model the relationship between phenotype and genotype data. Digital images of foliar disease represent complex multidimensional data, where each pixel has a color and intensity that is in-part influenced by the host’s genetics leading to resistance or susceptibility. Under uniform and controlled conditions, image-based phenotyping can capture variations that are driven primarily by the genetic background of the host. In this review, we have discussed methods of image segmentation to reduce the complexity of image data to a single phenotypic value for univariate GWAS analysis. With increasingly sophisticated AI models and more powerful computer hardware being released each year, it is plausible that the future of association genetics may involve feeding multimodal data (whole digital images of diseased leaves and entire genotype matrices) into deep learning models to derive complex nonlinear relationships beyond conventional GWAS. The concept has recently been explored in the medical field where CNN models were trained to correlate whole brain magnetic resonance images (MRI) to high-density genotype data to find SNPs associated with brain-related traits and disease (Yu et al. 2024). This approach of image-based association genetics modeling could also greatly benefit trait dissection and QTL mapping in advanced biparental mapping populations or in multi-parent advanced generation intercross (MAGIC) populations, particularly where chromosome scale assemblies are available from the parental founding lines.

The integration of advanced genebank foliar phenomics, genomic resources, and AI represents a transformative opportunity for association genetics that will allow researchers and breeders to unlock underutilized or novel QR genes and alleles from genebank germplasm, paving the way for more resilient cereal crops and sustainable agricultural practices. To realize this potential, the research community must prioritize open-access data sharing to assess traditional statistical modeling approaches used in association genetics against AI-based methods and collaborate as cross-disciplinary teams to bridge the gap between computer science and genetic research.
